# Detection of CNV in the *SH3RF2* gene and its effects on growth and carcass traits in chickens

**DOI:** 10.1186/s12863-020-0831-z

**Published:** 2020-02-28

**Authors:** Zhenzhu Jing, Xinlei Wang, Yingying Cheng, Chengjie Wei, Dan Hou, Tong Li, Wenya Li, Ruili Han, Hong Li, Guirong Sun, Yadong Tian, Xiaojun Liu, Xiangtao Kang, Zhuanjian Li

**Affiliations:** 1 Department of Animal genetics and breeding, College of Animal Science and Veterinary Medicine, Henan Agricultural University, Zhengzhou, 450046 Henan China; 2 Henan Innovative Engineering Research Center of Poultry Germplasm Resource, Zhengzhou, 450002 Henan China

**Keywords:** Chicken, *SH3RF2* gene, CNV, Growth traits, Carcass traits, Association analysis

## Abstract

**Background:**

The *SH3RF2* gene is a protein-coding gene located in a quantitative trait locus associated with body weight, and its deletion has been shown to be positively associated with body weight in chickens.

**Results:**

In the present study, CNV in the *SH3RF2* gene was detected in 4079 individuals from 17 populations, including the “Gushi ×Anka” F2 resource population and populations of Chinese native chickens, commercial layers, and commercial broilers. The F2 resource population was then used to investigate the genetic effects of the chicken *SH3RF2* gene. The results showed that the local chickens and commercial layers were all homozygous for the wild-type allele. Deletion mutation individuals were detected in all of the commercial broiler breeds except Hubbard broiler. A total of, 798 individuals in the F2 resource group were used to analyze the effects of genotype (DD/ID/II) on chicken production traits. The results showed that CNV was associated with 2-, 6-, 10-, and 12-week body weight (*P* = 0.026, 0.042, 0.021 and 0.039 respectively) and significantly associated with 8-week breast bone length (*P* = 0.045). The mutation was significantly associated with 8-week body weight (*P* = 0.007) and 4-week breast bone length (*P* = 0.010). CNV was significantly associated with evisceration weight, leg muscle weight, carcass weight, breast muscle weight and gizzard weight (*P* = 0.032, 0.033, 0.045, 0.004 and 0.000, respectively).

**Conclusions:**

CNV of the *SH3RF2* gene contributed to variation in the growth and weight gain of chickens.

## Background

In recent years, with the development of the economy and the improvement of people’s living standards, consumer requirements regarding the quality of poultry products, especially flavor and taste, have increased. Chinese local chickens have excellent characteristics, such as tender meat, good taste and unique flavor, which are favored by consumers. However, as growth traits and carcass traits are the main economic traits of poultry, the slow growth rates and low feed utilization rates of local chicken breeds in China represent limits to production. Therefore, genetic improvement through activities such as cultivating new varieties to increase the growth rate and the rate of lean meat gain in chickens has been a focus of research [1]. Thus, the use of modern molecular markers for marker-assisted selection and molecular breeding of chickens is important.

DNA molecular marker technology uses the gene library of the organism of interest without compromising the composition or expression of the genes [2]. It is a kind of genetic marker technology that can be used to identify variation at the nucleic acid level that potentially reflects functional differences among individuals. Molecular marker technology is helpful in revealing differences in the composition or arrangement of the whole genome or variation at the nucleotide level within a gene, providing insight into DNA variability and polymorphism. It can also be used to identify individuals containing target genes by genotype analysis of closely linked genetic markers of target genes, which can help improve selection efficiency (e.g., by reducing blind search) and accelerate the breeding process [3]. Compared with the use of traditional genetic markers, molecular marker-assisted selection (MAS) provides many marker loci, a large amount of genetic information, and strong repeatability of experiments, and it is not susceptible to environmental impacts and has no limitations regarding sex and age. Therefore, MAS allows early selection, shortens the generation interval, improves the selection intensity, and thus improves the efficiency and accuracy of selection. Due to these advantages, MAS has broad application prospects for animal genetic improvement [4]. At present, the application of DNA molecular marker technology in poultry genetic breeding mainly comprises genetic diversity analysis, germplasm identification, genetic relationship research, genetic map construction, quantitative trait loci (QTL) mapping, genome wide association study (GWAS) and molecular marker-assisted breeding [5]. A large number of genetic polymorphisms, including single nucleotide polymorphisms (SNPs), insertions/deletions (indels) and copy number variation (CNV), have been revealed in many species through whole-genome sequencing [6, 7]. CNV is an important source of genetic variation [8]. CNV is the main form of genome structural variation, which refers to the insertion, deletion, duplication, translocation and derived chromosome structural variation of DNA fragments larger than 1 kb in the genome relative to the reference sequence of the genome [9]. Because many CNVs contain entire genes, they are more difficult to identify and type than SNPs and indel copy number variants. As a result, they affect organisms to a greater extent than do these other types of polymorphisms. CNV is an important source of genetic variation complementary to SNP. CNV is associated with not only disease and abnormal development in livestock and poultry but also physical appearance and many economic traits [10–13]. For example, Wright [14] found that the first intron CNV of the *SOX5* gene is related to crown type. The bean crown mutation in the chicken is a dominant mutation, which greatly reduces the size of the bean crown, thereby reducing heat loss and preventing frostbite, and it is an adaptive characteristic of the chicken in a cold environment [14]. Dorshorst [15] found that the insertion of the *endothelin 3* (*EDN3*) gene was the main cause of overstaining of the skin of the black chicken. Elferink [16] found that a segment comprising a chicken prolactin receptor in the Z-chromosome is associated with the growth of the chickenundefineds fast-and-slow feather. Gorla [17] provided that a chicken CNV map based on the 600 K SNP chip array jointly with a genome-wide gene copy number estimates in a native chicken population. Other reports have shown two copy number variable genes associated with Marek’s disease, namely frizzled family receptor 6 (*FZD6*) and LIM and senescent cell antigen-like domain 1 (*LIMS1*) [18, 19]. These findings indicate that CNV in a gene can have important influences on economic characteristics of poultry and enrich organismal diversity. To date, few studies have investigated the effects of CNV in the SH3 domain containing ring finger 2 (*SH3RF2*) gene on poultry growth and development. Rubin [20] resequenced the chicken genome and found a selective clearance region in many chicken genomes that included the *SH3RF2* gene mutation site, which is located on chromosome 13, with all exons except the first exon being deleted. The total deletion length was 18,961 bp, and the gene was located in a body-related quantitative trait locus (QTL) range. The results of the study showed that the deletion mutation in the gene was positively associated with chicken body weight and fixed in the high-growth line of the broiler, appearing at lower frequencies in low-growth lines and commercial broilers, indicating that this deletion is closely related to broiler growth and body weight. Zhao [21] studied the frequency distribution of the indel mutation of the *SH3RF2* gene in 15 local chicken species in China and found no local chickens with the deletion mutation. In this study, we tested the CNV genotypes of local chicken breeds, commercial broilers, commercial laying hens and the “Gushi × Anka” chicken F2 resource population to identify *SH3RF2* gene mutations in the population and to analyze the association between CNV in this gene and chicken growth performance. The relationships between the CNV and growth and carcass traits of F2 resources in “Gushi × Anka” chickens showed that the *SH3RF2* gene can be used as an effective marker for chicken production traits.

## Results

### CNV genotyping

**Fig. 1:**
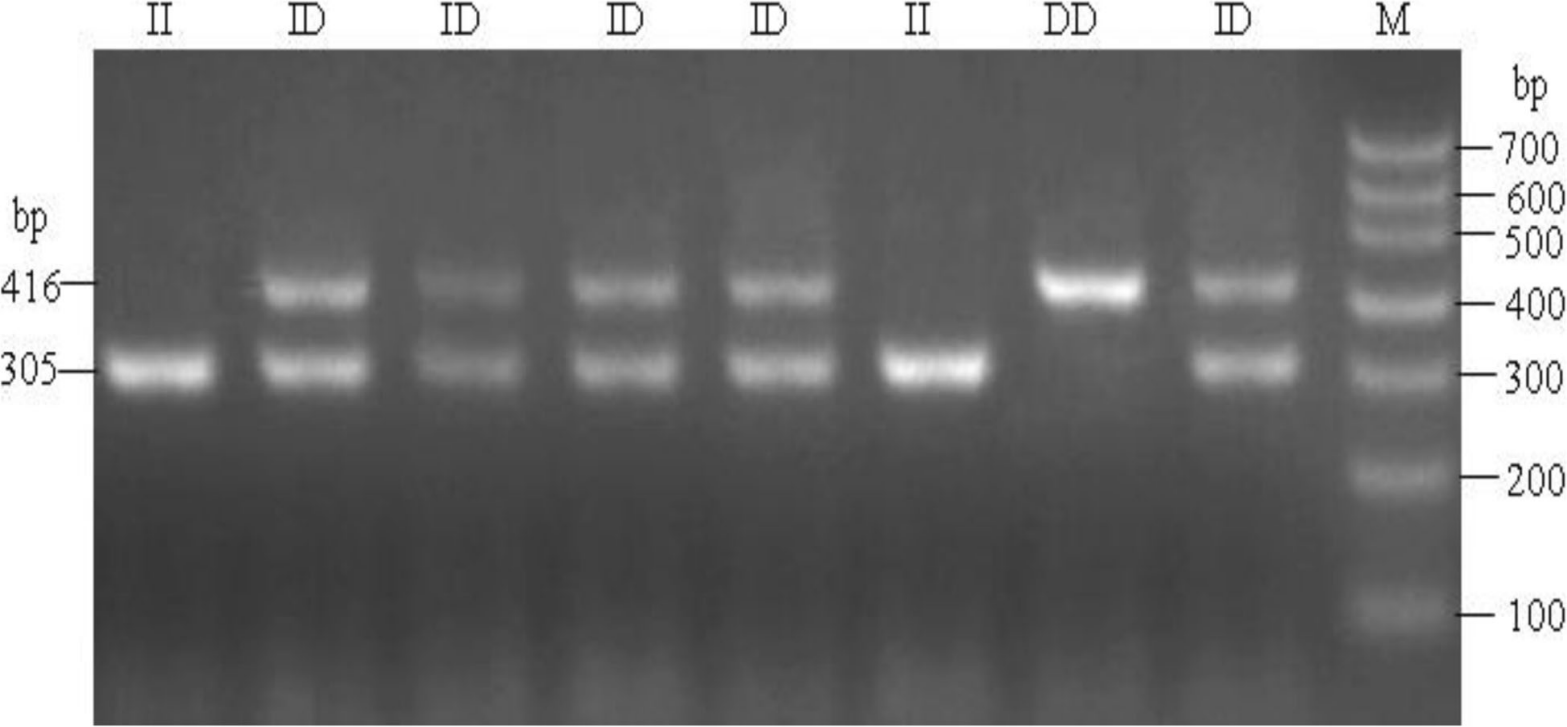
Agarose gel electrophoresis of *SH3RF2* gene mutation

After amplification, the “ins” (I) allele yielded only one 305 bp fragment and was determined to be a nondeletion allele; individuals homozygous for this allele were assigned the II genotype. The “del” (D) allele yielded only one 416 bp fragment and was determined to be a deletion allele; homozygous individuals were assigned the DD genotype. Individuals exhibiting both 305 bp and 416 bp product bands were considered heterozygous and assigned the ID genotype (Fig. 1).

### Analysis of the genetic parameters of *SH3RF2* gene mutations in different populations

The distribution of *SH3RF2* gene CNV genotypes and the gene frequencies in 4079 individuals from 17 populations were analyzed in this study (Additional file 1: Table S1). As shown in Table 1, there was a significant difference in genotypic frequency among the F2 resource population, AA broilers, R308 and CB broilers. ID genotypes and II genotypes were the predominant genotypes. The frequency of allele I was greater than that of allele D, and the frequency of II genotypes was higher than the frequencies of ID genotypes and DD genotypes in each population. The frequency of the DD genotype was highest in the AA broiler group. The expected heterozygosity (He) and number of allele (Ne) values were 0–0.35 and 1.00–1.55, respectively. According to the standard polymorphism information and polymorphism information content (PIC), PIC> 0.50 represents high polymorphism, PIC 0.25–0.50 represents moderate polymorphism, and PIC< 0.25 represents low polymorphism. The F2 resource group and AA broiler group showed moderate polymorphism, whereas the remaining populations showed low polymorphism.

**Table 1:** Genotypic and allelic frequencies and related genetic parameters for the chicken *SH3RF2* gene

Breed	Genotypic and allelic frequencies					He	Ne	PIC
	DD	ID	II	D	I			
F2/798	0.06	0.34	0.60	0.23	0.77	0.35	1.55	0.29
AA/636	0.31	0.28	0.41	0.45	0.55	0.49	1.98	0.37
R308/238	0.01	0.32	0.67	0.13	0.87	0.22	1.40	0.25
CB/193	0.16	0.01	0.83	0.17	0.91	0.09	1.10	0.15
HBD/282	0.00	0.08	0.92	0.04	0.96	0.08	1.08	0.07
B817/83	0.00	0.30	0.70	0.15	0.85	0.26	1.34	0.22
RW/187 XC/260	0.00 0.00	0.00 0.00	1.00 1.00	0.00 0.00	1.00 1.00	0.00 0.00	1.00 1.00	0.00 0.00
GS/174	0.00	0.00	1.00	0.00	1.00	0.00	1.00	0.00
CS/144	0.00	0.00	1.00	0.00	1.00	0.00	1.00	0.00
GF/18	0.00	0.00	1.00	0.00	1.00	0.00	1.00	0.00
YY/44	0.00	0.00	1.00	0.00	1.00	0.00	1.00	0.00
LS/202	0.00	0.00	1.00	0.00	1.00	0.00	1.00	0.00
WH/216	0.00	0.00	1.00	0.00	1.00	0.00	1.00	0.00
GC/88	0.00	0.00	1.00	0.00	1.00	0.00	1.00	0.00
HL/456	0.00	0.00	1.00	0.00	1.00	0.00	1.00	0.00
LB/60	0.00	0.00	1.00	0.00	1.00	0.00	1.00	0.00

F2, AA, R308, CB, HBD, B817, RW, XC, GS, CS, GF, YY, LS, WH, GC, HL, and LB denote F2 resource population, Arbor Acres broiler, Ross308, Cobb broiler, Hubbard broiler, 817 broiler, Recessive white chicken, Xichuan chicken, Gushi chicken, Changshun chicken, Guifei chicken, Yunyang chicken, Lushi chicken, Wuhei chicken, Hy-line brown hen and Lohmann brown laying hen, respectively

### Association analysis of *SH3RF2* genotype and growth traits in the F2 resource population

As shown in Table 2, the growth traits of the F2 generation were significantly associated with *SH3RF2* genotype. Genotype was significantly associated with BW at 2, 6, 8, 10 and 12 weeks (*P* < 0.05), BBL at 8 weeks (*P* < 0.05), 8-week BW (*P* < 0.01) and 4-week BBL (*P* < 0.01). The growth trait values of the homozygous genotype DD were higher than those of the other two genotypes each week. The 8-week BW and 4-week BBL of individuals with the DD genotype were 92.3 and 97.2% higher, respectively, than those of individuals with the II genotype, indicating that the DD genotype is an economically dominant genotype. The growth trait values of the ID genotype were higher than those of the II genotype each week, indicating that allele D was the dominant allele.

**Table 2:** Association analysis of CNV in the *SH3RF2* gene and growth traits in the F2 resource population

Traits	Mean ± SE			*P*-value
	II (*n* = 482)	ID (*n* = 269)	DD (*n* = 47)	
BW2 (g)	115.985 ± 2.855^ab^	122.347 ± 1.162^a^	123.847 ± 0.861^a^	0.026
BW4 (g)	308.965 ± 6.814	322.282 ± 2.814	324.787 ± 2.115	0.081
BW6 (g)	536.205 ± 12.649^ab^	563.207 ± 5.306^a^	569.033 ± 3.984^a^	0.042
BW8 (g)	762.844 ± 19.211^b^	817.485 ± 7.894^a^	826.332 ± 5.989^a^	0.007
BW10 (g)	1055.384 ± 23.335^ab^	1113.946 ± 9.888^a^	1123.156 ± 7.358^a^	0.021
BW12 (g)	1287.379 ± 27.936^ab^	1358.504 ± 11.755^a^	1361.616 ± 8.84^a^	0.039
SL8 (cm)	7.713 ± 0.094	7.923 ± 0.039	7.947 ± 0.029	0.061
CW8 (cm)	5.548 ± 0.082	5.689 ± 0.034	5.698 ± 0.025	0.215
SG8 (cm)	3.377 ± 0.033	3.411 ± 0.014	3.439 ± 0.01	0.079
BBL4 (cm)	6.061 ± 0.074^ab^	6.193 ± 0.031^a^	6.267 ± 0.023^a^	0.010
BBL8 (cm)	8.722 ± 0.097^ab^	8.908 ± 0.04^a^	8.964 ± 0.03^a^	0.045
BSL4 (cm)	11.154 ± 0.118^ab^	11.347 ± 0.048^a^	11.475 ± 0.036^a^	0.008

BW0, BW2, BW4, BW6, BW8, BW10, and BW12 denote body weight at 0 days, 2 weeks, 4 weeks, 6 weeks, 8 weeks, 10 weeks and 12 weeks, respectively. *BW* Body weight, *SL* Shank length, *CW* Chest width, *SG* Shank circumference, *BBL* Breastbone length, *BSL* Body slanting length. The same letters within a row indicate no significant difference (*P* > 0.05); different letters indicate significant differences (*P* < 0.05)

### Association analysis of *SH3RF2* genotype and carcass traits in the F2 resource population

The association analysis showed that *SH3RF2* genotype was significantly related to SEW, EW, SEP, EP, LMW, LWR, GWR and CW (*P* < 0.05) and MW, GW and BMWR (*P* < 0.01). Table 3 shows that the slaughter index of the different genotypes followed the order DD genotype > ID genotype > II genotype, which is consistent with the growth trait results.

**Table 3:** Association analysis of CNV in the *SH3RF2* and carcass traits in the F2 resource population

Traits	Mean ± SE			*P*-value
	II (*n* = 482)	ID (*n* = 269)	DD (*n* = 47)	
EW (g)	870.526 ± 20.885^ab^	922.81 ± 8.717^a^	928.011 ± 6.63^a^	0.032
GW (g)	25.955 ± 0.674^b^	27.568 ± 0.284^b^	28.450 ± 0.214^ab^	0.000
CW (g)	1134.15 ± 24.818^ab^	1190.264 ± 10.44^a^	1198.651 ± 7.936^a^	0.045
MW (g)	63.095 ± 2.21b	71.055 ± 0.918a	71.3 ± 0.695^a^	0.002
SEW (g)	1046.01 ± 24.113^ab^	1104.431 ± 10.102^a^	1109.572 ± 7.62^a^	0.042
LMW (g)	141.966 ± 3.465^ab^	149.74 ± 1.464^a^	151.338 ± 1.103^a^	0.033
EP (%)	67.374 ± 0.288^ab^	67.829 ± 0.121^a^	68.109 ± 0.092^a^	0.019
SEP (%)	81.075 ± 0.288^a^	81.109 ± 0.122^ab^	81.54 ± 0.092^a^	0.011
GWR (%)	2.016 ± 0.045^a^	2.053 ± 0.019^ab^	2.113 ± 0.015^a^	0.012
HWP (%)	3.311 ± 0.048	3.186 ± 0.02	3.208 ± 0.016	0.059
LWR (%)	2.246 ± 0.045^a^	2.125 ± 0.019^ab^	2.145 ± 0.015^a^	0.048
BMWR (%)	14.383 ± 0.267^b^	15.346 ± 0.11^a^	15.233 ± 0.085^a^	0.004
BWLP (%)	22.687 ± 0.745	23.738 ± 0.313	24.342 ± 0.238	0.053

*EW* Evisceration weight, *GW* Gizzard weight, *CW* Carcass weight, *MW* Breast muscle weight, *SEW* Semievisceration weight, *LMW* Leg muscle weight, *EP* Evisceration percentage, *SEP* Semievisceration weight rate, *GWR* Gizzard weight rate, *HWP* Head weight percentage, *LWR* Liver weight rate, *BMWR* Breast muscle weight rate, *BWLP* Breast muscle water loss rate. The same letters within a row indicate no significant difference (*P* > 0.05); different letters indicate significant differences (*P* < 0.05)

## Discussion

### Analysis of *SH3RF2* gene polymorphism and the genetic population structure of different breeds of chickens

The genetic resources of poultry species in China are diverse and contain great genetic variation and selection potential [22]. The vigorous development of animal husbandry will promote breeding efforts to improve the performance of chicken production [23]. Therefore, it is necessary to study the genetic variation of different chicken breeds. In this study, the frequencies of the DD genotype and D allele were lower than the other genotypes and I allele, respectively, in the studied populations, and few individuals with deletions were found among the populations of local and commercial layers. Whereas moderate polymorphism was found in the F2 resource population and the AA broiler, the remaining breeds showed low polymorphism. According to the polymorphism information content standard, this result indicates that the F2 resource population and AA broilers have greater selection potential in breeding and growth development efforts. In general, allele frequency reflects genetic diversity between populations. We found that allele D was the dominant allele in each population, suggesting that the gene did not undergo the same selection in the evolution of the different breeds of chicken. Moreover, artificial selection has a significant impact on the number of genes and distribution of genetic variation in different varieties [24]. We found that the frequency of DD genotypes was higher in commercial broiler populations with fast, large and high yields than that in other breeds, indicating that targeted breeding of commercial broiler chickens promoted the fixation of DD genotypes, which may be related to the rapid growth of commercial broiler chickens. We found no mutant deletion in commercial layers, which may be related to the selective breeding of layers.

### Associations between the *SH3RF2* gene mutation and growth and carcass traits

Body weight and body size are important indicators of the body development of poultry and are closely related to important economic characteristics [25]. To date, few studies on the *SH3RF2* gene in poultry production have been conducted. Rubin [20] analyzed 400 chickens from F8 generations of high-growth and low-growth lines and found that the *SH3RF2* gene deletion mutation had highly significant effects on chicken growth traits (*P* < 0.01). In the present study, the F2 resource population of Anka and Gushi chickens was used to analyze the relationships between the *SH3RF2* gene mutation and growth and carcass traits of chickens. Consistent with the previous study, the results showed that the mutation had significant influences on the growth and carcass traits of chickens. As poultry muscles are mainly distributed in the chest and legs, the yield of chest and leg muscles is an important factor determining the slaughtering performance of poultry and is one of the traits considered in poultry breeding [26]. In this study, *SH3RF2* genotype was significantly associated with growth traits, such as BW and BBL, and slaughter indicators, such as GW, GW, LMW and CW (*P* < 0.05). In addition, individuals with the DD genotype had higher phenotype values than did individuals with II genotypes or ID genotype. The results show that the DD genotype has a significant dominant effect on traits of the F2 resource population.

## Conclusion

This study found that CNV in the *SH3RF2* gene was related to most of the studied growth traits and carcass traits in chickens. The results show that the mutation can be used for MAS breeding in chickens. Molecular breeding of chickens can reduce breeding costs, shorten the generation gap, and improve the efficiency of breeding to provide technical support for local chicken breeding in China.

## Methods

### Experimental animals

To identify the distributions of the different genotypes in different populations, individuals of the Gushi-Anka chicken F2 generation resource population, 8 local varieties, 5 chicken varieties and 2 layer varieties were investigated, with DNA samples collected from a total of 4079 individuals (Table 4). All blood samples were collected through the wing vein, after which 1:300 multidimensional hormone was used to reduce the stress response. Whole genome DNA was extracted from whole blood using the phenol-chloroform method. All chickens used in the study were healthy animals raised in the same environment with ad libitum access to feed and water. To produce the F2 resource population, two hatchings were obtained from an F1 generation constructed via reciprocal crossing between Chinese native Gushi chickens (representing a slow-growing Chinese native chicken) and Anka broilers (representing a fast-growing broiler). Then according to a 1:9 ratio of males to females, an F2 generation was produced by mating the offspring with other family hens. The hens represented 7 families. The F2 generation was thus composed of 7 families, with Anka chicken as the male parent of 4 orthogonal lines and Gushi chicken as the male parent of the 3 reverse cross lines. We used an F2 resource family as previously described by Liang et al. [27] and Li et al. [28].

**Table 4:** Information on the studied chicken breeds

Breed	n	Characteristic	Breed	n	Characteristic
F2	798	segregating population	XC	260	slow-growing, dual-type
AA	636	fast-growing, meat-type	GS	174	slow-growing, dual-type
R308	238	fast-growing, meat-type	CS	144	slow-growing, dual-type
HBD	282	fast-growing, meat-type	LS	202	slow-growing, dual-type
CB	193	fast-growing, meat-type	YY	44	slow-growing, dual-type
B817	83	fast-growing, meat-type	WH	216	slow-growing, dual-type
HL	456	egg-type	GF	18	slow-growing, dual-type
LB	60	egg-type	GC	88	slow-growing, game-type
RW	187	slow-growing, dual-type			

F2, AA, R308, HBD, CB, B817, HL, LB, RW, XC, GS, CS, LS, YY, WH, GF, and GC denote F2 resource population, Arbor Acres broiler, Ross308 broiler, Hubbard broiler, Cobb broiler, 817 broiler, Hy-line brown hen, Lohmann brown laying hen, Recessive white chicken, Xichuan chicken, Gushi chicken, Changshun chicken, Lushi chicken, Yunyang chicken, Wuhei chicken, Guifei chicken, and Henan gamecock, respectively. “dual-type” denotes meat-egg-type

In henan provincial poultry germplasm resources innovation engineering research center, Gushi-Anka F2 resource population were housed in cages with 50 chickens per cage at 1 d, providing 392 cm^2^ per bird. At the age of 8-week, it was transferred to three birds per cage (448 cm^2^ / bird). A total of 798 individuals from the F2 resource population were euthanised at 84 d. In the laboratory of Henan Agricultural University, 5% Pentobarbital 2 mL (No. 57–33-0 of Chinese Academy of Sciences, Beijing Siyuan Technology Co., Ltd.) was injected intraperitoneally into the Gushi-Anka F2 resource population. After 2–3 min of no spontaneous respiration, carotid artery bleeding occurred. Intraperitoneal injection of pentobarbital sodium can lead to rapid and relatively stress-free death. From hatch to slaughter, several chicken growth traits, including body weight and body size indexes, were measured. Each chicken was weighed every 2 weeks. Shank length was measured at 0, 4, 8 and 12 weeks, and shank girth, chest depth, breast bone length, body slanting length and pelvis breadth were determined at 4, 8 and 12 weeks. Descriptions of the construction of the F2 population, feeding management and trait determination procedures can be found in a previous study [29, 30]. Information on 17 populations is presented in Table 4.

### Primer and PCR amplification

The primers used in this study were all designed by Rubin [19] (Forward: 5′-TGCTTCGGGCTGAGCCTTCT-3′, Reverse1: 5′-CGCCCAAGCTGTGTCCT-3′, Reverse2: 5′-CTGTCGGGCACGTGAGTGAA-3′). Assays were performed by PCR in a total volume of 10 μL containing 5 μL of 2 × *Taq* Master Mix (Kangwei, Beijing, China), 0.5 μL of forward primer, 0.5 μL of reverse primer, 2.5 μL of ultrapure water and 1 μL of genomic DNA. The PCR amplification was performed as follows: 95 °C for 5 min followed by 30 cycles at 95 °C for 30 s, 64 °C for 30 s, and 72 °C for 10 min. The samples were then chilled at 4 °C. To determine genotype, aliquots from each reaction (7 μL) were subjected to electrophoresis in a 2% agarose gel.

### Statistical analysis

Statistical analyses of the associations between genotype and the selected traits of the F2 chickens were performed using IBM SPSS (SPSS for Windows, Standard version 24; SPSS, USA). Genotype effects were analyzed by a multivariate linear model, and differences among genotypes were evaluated by Bonferroni’s multiple comparison method. The analyses followed a previous study [31]. All data for each trait obtained by statistical analysis are presented as the mean ± standard error (mean ± SE). *P* < 0.05 was considered to indicate statistical significance [32].

## Supplementary information

**Additional file 1: Table S1.** The Genotypic information on the studied chicken breeds. (XLS 234 kb)

## Data Availability

All the data supporting the conclusions of the study are included in the manuscript and Additional file 1.

## Abbreviations

AA: Arbor Acres broiler
B817: 817 broiler
BBL: Breastbone length
BMWR: Breast muscle weight rate
BSL: Body slanting length
BW: Body weight
BWLP: Breast muscle water loss rate
CB: Cobb broiler
CHW: Chest width
CNV: Copy number variation
CS: Changshun chicken
CW: Carcass weight
EDN3: Endothelin 3
EP: Evisceration percentage
EW: Evisceration weight
F2: F2 resource population
FZD6: Frizzled family receptor 6
GC: Henan gamecock
GF: Guifei chicken
GS: Gushi chicken
GW: Gizzard weight
GWAS: Genome wide association study
GWR: Gizzard weight rate
HBD: Hubbard broiler
He: Expected heterozygosity
HL: Hyline brown hen
HWP: Head weight percentage
Indels: Insertions/deletions
LM: Lohmann brown laying hen
LMW: Leg muscle weight
LS: Lushi chicken
LWR: Liver weight rate
MAS: Marker-assisted selection
MW: Breast muscle weight
Ne: Number of allele
PCR: Polymerase chain reaction
PIC: Polymorphism information content
QTL: Quantitative trait loci
R308: Ross308 broiler
RW: Recessive white chicken
SE: Standard error
SEP: Semievisceration weight rate
SEW: Semievisceration weight
SG: Shank circumference
SH3RF2: SH3 domain containing ring finger 2
SL: Shank length
SNPs: Single nucleotide polymorphisms
WH: Wuhei chicken
XC: Xichuan chicken
YY: Yunyang chicken
